# Preparation and Dielectric Properties of K_1/2_Na_1/2_NbO_3_ Ceramics Obtained from Mechanically Activated Powders

**DOI:** 10.3390/ma13020401

**Published:** 2020-01-15

**Authors:** Izabela Szafraniak-Wiza, Jolanta Dzik, Dariusz Bochenek, Diana Szalbot, Małgorzata Adamczyk-Habrajska

**Affiliations:** 1 Institute of Materials Science and Engineering, Poznań University of Technology, Jana Pawła II 24, 61-138 Poznań, Poland; izabela.szafraniak-wiza@put.poznan.pl; 2 Faculty of Science and Technology, University of Silesia, Institute of Materials Engineering, 12, Zytnia Str., 41-200 Sosnowiec, Poland; jolanta.dzik@us.edu.pl (J.D.); dariusz.bochenek@us.edu.pl (D.B.); diana.szalbot@us.edu.pl (D.S.)

**Keywords:** lead-free materials, perovskites, mechanical activation, potassium sodium niobate, dielectric properties

## Abstract

Alkaline based materials have been considered as a replacement for environmentally harmful Pb(Zr,Ti)O_3_ (PZT) electro-ceramics. In this paper, the K_1/2_Na_1/2_NbO_3_ (KNN) ceramics were prepared in a three stage process: first Nb_2_O_5_, Na_2_CO_3,_ and K_2_CO_3_ were milled in a high energy mill (shaker type) for different periods, between 25 h and 100 h, consecutively a solid state reaction was carried out at 550 °C. Finally, the uniaxially pressed samples were sintered at 1000 °C. The reaction temperature is lower for mechanically activated powders than in the case of the conventional solid-state method. The ceramic samples, prepared from the mechanically activated powders, were investigated by dielectric spectroscopy. The influence of the duration of the mechanical activation on the properties of the ceramic materials, e.g., ceramic microstructures, phase transition temperatures, character of the temperature dependences of dielectric permittivity, are discussed.

## 1. Introduction

Commercially produced piezoelectric ceramics, used as actuators, transducers, sensors and energy harvesters, are PZT-based (Pb(Zr,Ti)O_3_) materials [1,2]. Due to the high content of lead and the negative impact thereof on humans and the environment a lead-free alternative has been sought [1,3]. Therefore, lead-free piezoelectric ceramics have become an important research topic in recent years [3]. (K,Na)NbO_3_ (KNN) solid solution is a very promising candidate to replace PZT. High Curie temperature, biocompability, and a high electromechanical coupling factor make this material very attractive, nevertheless the possibility of obtaining satisfactory properties of KNN is strongly dependent on the processing conditions [4]. Several problems occur during the conventional solid- state synthesis, mainly due to the evaporation of alkali elements at high temperatures. Furthermore, conventional KNN ceramics exhibit poor densification and anomalous grain growth. In order to obtain well compacted, fine-grained ceramics with proper stoichiometric composition, it is necessary to modify the technological route or to use powders with higher sinterability. One of the possible ways to improve sinterability is the lowering of the processing temperature and preventing the evaporation of alkali. Therefore, the mechanochemical synthesis seems to be very suitable for the preparation of perovskite materials, due to the fine grains and processing at room temperature. Many perovskites can be obtained directly after high energy ball milling at room temperature [5,6], however, successful synthesis of KNN through the mechanochemical method has not been reported, even for a very long procedure (up to 30 days) and also for very high energy impacts during milling [7,8]. Milling (or high energy ball milling) has also been used to produce fine grain powders from the synthesised KNN materials [9,10].

In this way, first, the KNN materials were obtained by conventional solid-state reaction methods. Consecutively the powders were milled in order to obtain nanocrystalline grains. The existence of nanograins resulted in better compacting properties, and consequently allowed better sintered ceramics to be produced. Another interesting possibility based on a high energy milling treatment is mechanochemical activation. In this process, the starting powders before calcination (solid-state reaction) are milled to improve the homogeneity of the raw materials, reduce the particle size of the grains and to create crystal defects. Consequently, the reactivity of the mixture increases, which generates a lower temperature of sintering. Moreover, the mechanochemically activated mixtures often are of a very fine size (usually in the nanoscale range), which makes it possible to improve the density of ceramics. In the preparation of high-quality electroceramic materials, of equal importance are the powder preparation and the method of powder compaction. For densification, free sintering and hot pressing have been used for many years. However, obtaining advanced nanostructured materials requires the use of more advanced technologies, such as spark plasma sintering (SPS) and hybrid flash spark plasma sintering. SPS involves direct joule heating of electrically conductive dies (usually graphite), whereas the more advanced hybrid spark SPS employs a thermal runaway to achieve ultrafast sintering with heating rates as high as 10,000 °C/min [11,12,13].

The mechanochemical activation approach has been applied for the preparation of KNN, as well as several aspects having been discussed, nevertheless, investigations of the influence of high energy milling times before the calcinations on the final properties of ceramics have not been addressed in detail [8,14,15,16]. The present work focused on the preparation and dielectric property investigations of K_1/2_Na_1/2_NbO_3_. The starting powders were milled for different periods in a high-energy mill. The microstructure of the KNN powders and ceramics was investigated by SEM, whereas the chemical homogeneity of the samples was evaluated by EDS analysis. Dielectric permittivity, as well as dielectric loss tangent as a function of temperature (RT–550 °C) and frequency (1 kHz–1 MHz), were determined by dielectric spectroscopy. The influence of high energy milling on the final properties of KNN ceramics, as well as the comparison of dielectric properties between the mechanically activated and the conventionally prepared KNN ceramics, is discussed.

## 2. Materials and Methods

KNN with the stoichiometry of K_1/2_Na_1/2_NbO_3_ was obtained from Na_2_CO_3_ (Sigma-Aldrich, St. Louis, MI, USA, purity ≥99.0%), K_2_CO_3_ (Sigma-Aldrich purity 99.99%), and Nb_2_O_5_ (Sigma-Aldrich purity 99.99%). The mixture of powders in the stoichiometric ratio was milled in a SPEX 8000 high-energy shaker-type mill. The powders were milled for different periods, between 25 h and 100 h. The ball to powder weight ratio (BPR) parameter was 6:1. The powder, obtained by milling for 50 h, was calcined at different temperatures, between 450 °C and 700 °C, for 2 h, in order to define the temperature of crystallization. On the basis of the investigation, all mechanically activated powders were calcined at 550 °C, for 2 h, in an air atmosphere. The calcined powders were pressed with steel dies into discs with a diameter of d = 10 mm and thickness of h = 1 mm, using a one-sided, uniaxial cold pressing method, and a hydraulic press, at a pressure of p = 30 MPa. The obtained pellets were conventionally free sintered at 1000 °C, for 3 h, in an air atmosphere. The density of the obtained ceramic materials changed from 4.35 g/cm^3^ to 4.38 g/cm^3^, which is 96.5% and 97.2% of the theoretical density, respectively [17].

XRD and SEM analyses were applied to monitor the crystallographic structures of materials after each preparation step. XRD measurements were performed with a PANalytical Empyrean X-ray powder diffractometer (CuKα radiation, 45 kV, 40 mA). The microstructures of the obtained powder samples and ceramics were examined with a (SEM) JEOL (Tokyo, Japan) JSM-7100 TTL LV Field Emission Scanning Electron Microscope, with an energy dispersive X-ray spectrometer (EDS). The microstructure observations were performed on the fractured surface of the sintered ceramics samples which were coated with gold to provide electrical conductivity and to avoid any charging effects. EDS was used for the studies of chemical homogeneity of the obtained ceramics.

The dielectric permittivity as a function of temperature was measured in a field of several frequencies, in the range of 0.1–1 MHz, by using a computerized automatic system based on an LRC meter (HP 4192A, Hewlett-Packard, Palo Alto, CA, USA).

## 3. Results and Discussion

**Figure 1:**
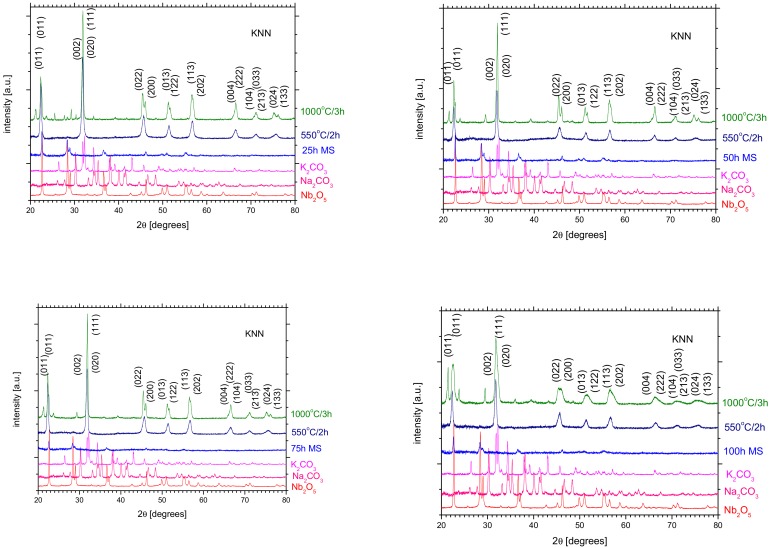
XRD diffraction patterns of powders at different stages of preparation.

The functional properties of the perovskite ceramics are strongly dependent on the preparation method and conditions thereof, as well as the final material crystallographic structures and microstructures related thereto. In the present work, the influence of mechanical treatment on the properties was investigated. First of all, the starting powders were milled for different durations. The XRD diffraction patterns of the milled materials are presented in Figure 1. The mixture after all milling periods exhibits visible traces of the crystalline structure originating from the starting powder of Nb_2_O_5_, as well as a relatively large amount of the amorphous material. Even after such a relatively long milling time of 100 h, the amorphization was not complete. As has been widely described in our previous paper [18], the analysis of the results of X-ray investigations clearly confirmed the presence of a single phase in the sample. All the lines visible on the X-ray diffraction patterns point to an orthorhombic type of structure. The position and intensity of the diffraction lines are in a good agreement with the ICDD (PDF-4) pattern 4-007-8808 determined for the KNbO_3_ phase. The model was used as a starter for the Rietveld refinement.

The Rietveld method allowed the determination the unit cell parameters and volume (V) of all investigated ceramics (Table 1).

The analysis of the values of the lattice parameters obtained for all samples indicated the lack of significant impact of the high energy milling time on the crystallographic structure. The a_0_ and b_0_ cell parameters do not show any visible tendency. Solely the c_0_ parameter slightly increases with the increasing milling time. As consequence, the lattice distortion in the direction [001] increases.

**Figure 2:**
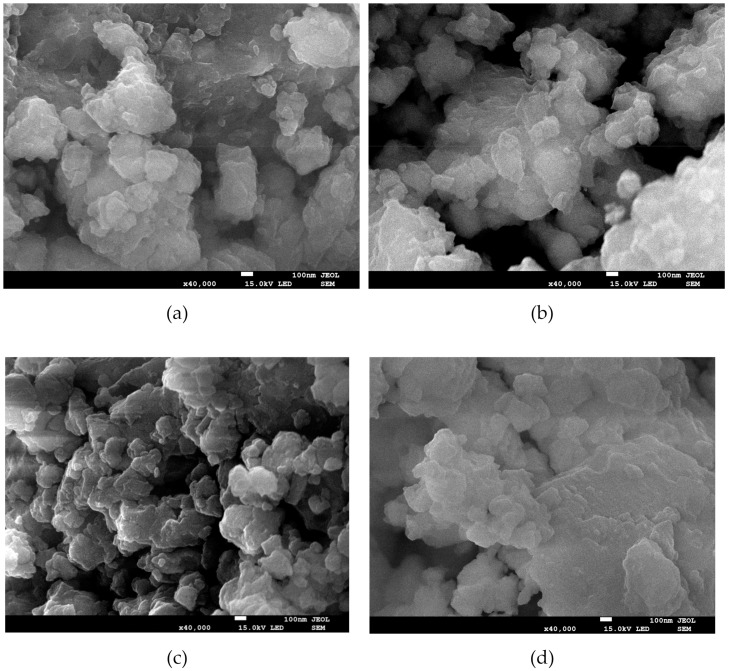
SEM micrographs of powders after 25 h (**a**), 50 h (**b**), 75 h (**c**), and 100 h (**d**) of high energy milling.

The SEM microstructures of the mechanically activated powders are presented in Figure 2. The powders, after different milling periods, exhibit a similar microstructure. The powders are built from irregular grains with a wide size distribution (from below 100 nm to above several microns). The edges of the grains are not sharp and are often rounded. The grains form irregular agglomerates, the size of which increases with increasing milling time. The presented results reveal that the prolonged milling time does not affect the powder morphology. This claim seemingly contradicts the observations of other authors [19,20]. Namely, the authors most often used relatively short milling times (for instance 2, 6 or 8 h) and observed a significant reduction in powder particles. However, in the case of (K_0.485_Na_0.485_Li_0.03_)(Nb_0.96_Sb_0.04_)O_3_ ceramics, the authors noted an increase in the particle size for longer milling time (longer than 20 h) [21]. Such a behavior is in line with the analytical model proposed by Gusev et al. [22]. The model proposed a very complicated relationship between milling time and the size of post milling particles. The relation predicts that an increase in the milling time leads to a gradual decrease in the powder particle size to the saturation value. The milling times used in the case of the discussed KNN ceramics were long enough to result in such a situation. The powder milled for sufficient time reaches critical conditions of instability with the accumulation of a significant amount of energy. The surplus energy in the smaller particles is a consequence of the increase of the specific surface energy of the particles, connected with the changes in the surface to volume ratio, as well as the microstrains produced in the particles. In this process of releasing excess energy, the smaller particles coalesce to form bigger ones and create agglomerates.

**Figure 3:**
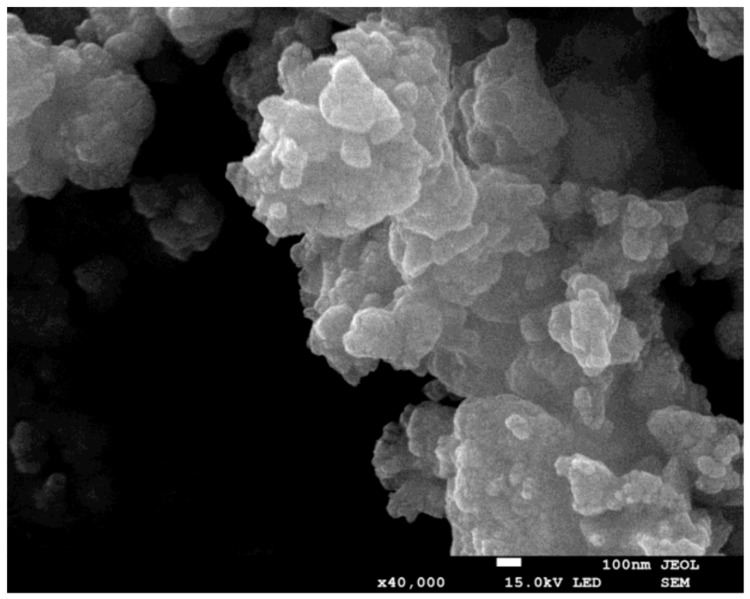
SEM image of the 100 h milled powder after crystallisation.

In order to form the perovskite phase, it is necessary to crystallize the powders at high temperature. Commonly for mechanically activated materials, the crystallization temperature is lower than in the case of conventional methods [7,8]. The powder milled for 25 h was annealed at different temperatures, between 450 °C and 700 °C, for 2 h. The development of the perovskite phase was monitored by XRD. On the basis of this thermal treatment, the crystallization of the mechanically activated powder was set at 550 °C. In order to compare the effect of the mechanochemical treatment, all powders were crystallized at 550 °C, for 2 h. The XRD investigation confirmed the formation of the perovskite phase. In all cases, the morphology of the powder did not change significantly (Figure 3). Only the size of the grains increased, whereas the smallest grains disappeared, which is connected with the development of agglomerates. As mentioned above, the longer milling time facilitates the creation of agglomerates. The proximity and adhesion of particles in agglomerates promotes matter displacement—atoms migrate from one grain towards another through the separating grain boundary generating grain growth [23].

**Figure 4:**
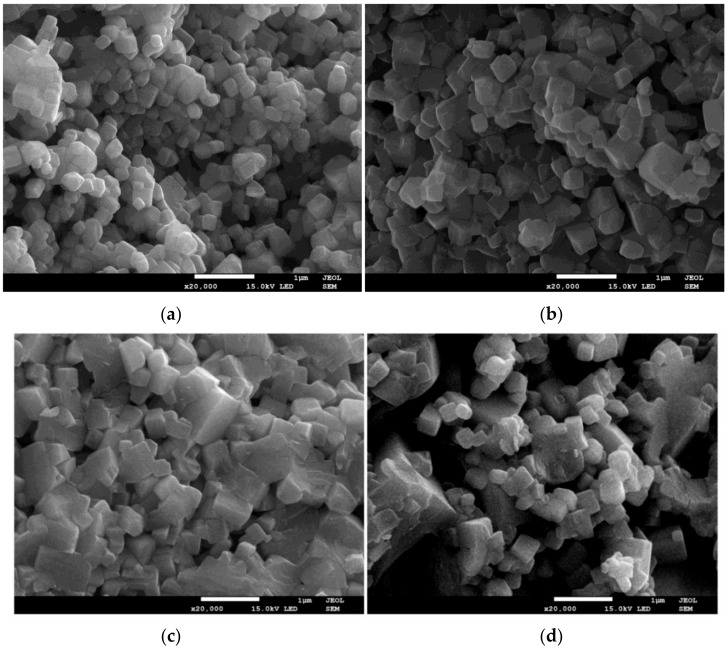
SEM micrographs of ceramics obtained from 25 h (**a**), 50 h (**b**), 75 h (**c**), and 100 h (**d**) milled powders.

In the next stage, the powders were unixially pressed into pellets and finally sintered at 1000 °C, for 3 h, in an air atmosphere. The typical SEM micrographs of sineterd ceramics are presented in Figure 4. The morphology is typical for KNN ceramics [3]. The microstructure consists of well-developed, cuboidal grains with slim grain boundaries. The mechanically activated ceramics are built of grains significantly smaller than in the case of ceramics obtained by the classical method. The average grain size of ceramics prepared by the conventional ceramics processing route is in the range between 1.36 µm and 2.17 µm, depending on the preparation conditions (time and temperature of the sintering process) [24,25]. The average grain size of the investigated ceramics is strongly dependent on the milling time, and varies from 170 nm for the ceramics milled for 25 h, to 470 nm for the ceramics milled for 100 h.

**Figure 5:**
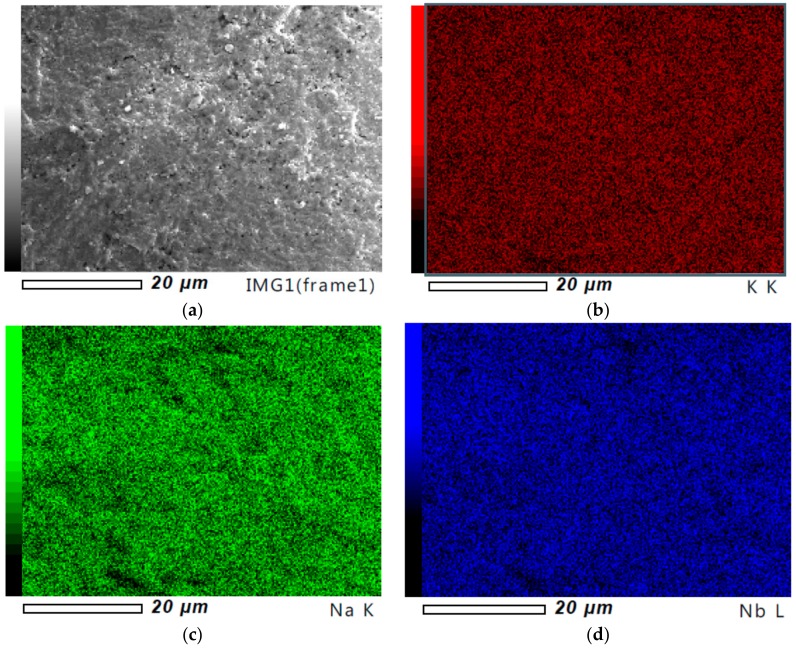
SEM micrographs of the ceramics obtained from the 75 h (**a**), element mapping of KNN ceramics across entire section–potasium(**b**), sodium (**c**) and niobium (**d**).

The distribution of individual elements within the grains was verified by using an energy dispersion X-ray spectrometer (EDS). The analysis indicated a homogenous distribution of all elements (Figure 5). Moreover, the quantative microanalysis showed that the obtained ceramics had a stochiometry close to the nominal one and the element content was not dependent on the milling time.

**Figure 6:**
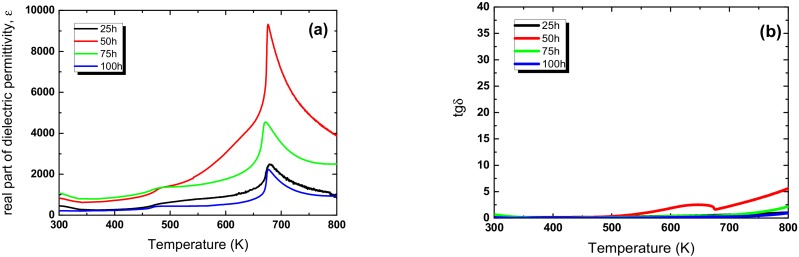
Temperature dependencies of dielectric permittivity (**a**) and loss tangent (**b**) measured during heating at frequency 10 kH for KNN ceramics obtained from 25 h, 50 h, 75 h, and 100 h milled powders.

The differences in grain size or grain/ceramic morphology usually have an influence on the dielectric and electric properties, e.g., such a behaviour has been observed for the KNN ceramics [26]. The temperature dependences of dielectric permittivity *ε*(T) and dielectric losses *tan δ*(T) are shown in Figure 6. The value of ε at room temperature for ceramics milled for 25 h is equal to ~450. The prolongation of the mechanochemical activation to 75 h resulted in an increase in the dielectric permittivity to ~1100, whereas for the ceramics obtained via the classical method, the value *ε* = 500 was recorded [27,28].

The *ε*(T) dependencies (Figure 6) exhibit two anomalies, namely that their *ε* value and their temperature depend on the milling time (Table 2). The first one, observed closer to room temperature, is related to the orthorhombic–tetragonal phase transition. The second one, with clear peak of dielectric permittivity, is related to the ferroelectric–paraelectric phase transition. The first anomaly is observed in the range of 480–490 K. The temperature of phase transitions T_OT_ increases with increasing milling time. The temperature of the second one (T_m_) appears in the range 671–679 K and decreases with the milling time.

Based on the KNN phase diagram, the phase transitions appear in the range of temperature 476–482 K (the first one) and between 677–691 K (the second one) for the K_1−x_Na_x_NbO_3_ ceramics with x = 0.48–0.54. The 6% fluctuation of sodium and potassium cause a 14 K change of temperature. In the case of the obtained ceramics, the difference of the phase transition temperatures cannot originate from stoichiometric difference, since EDS (within the error limits of method investigation) did not reveal the composition changes of the obtained ceramics related to the milling time. Therefore, the deviation of the phase transition temperatures cannot be connected directly to the stoichiometric fluctuations. The temperature changes may be related to the grain size effect.

Also the *ε*_max_ value significantly changes with the mechanochemical activation time, and achieves the highest level 9200 (at 10^4^ Hz) for the ceramics obtained from the powder milled for 25 h. The maximum value of *ε* for the ceramics obtained using the conventional method does not exceed 6000 (for 10^4^ Hz) [28]. Both of the observed changes, i.e., the anomaly and the maximum value of dielectric permittivity, can be explained by changes in the size of the grains of the ceramics. It is a commonly known effect referred to as the grain size effect. In some ceramics, for instance in the case of BaTiO_3_, a decrease in grain size causes an increase of the value of the dielectric permittivity, and this change is clearly visible for the maximum value [29,30]. The tendency is observed up to a certain critical size, below which the dielectric permittivity decreases. The threshold size for BaTiO_3_ ceramics is estimated to be approximately 800 nm [31,32]. The origin of the threshold size is not quite comprehensible. L.Curecherin et al. suggested that the changes in tendency were connected with an increase in the proportion of non-ferroelectric grain boundaries in the total sample volume [33]. According to Uchino et al. the tendency could be explainable on the basis of the surface tension stress for small particles [25,34]. The analysis of the correlation between the temperature dependencies of dielectric permittivity obtained for the investigated ceramics and the average grain size allowed the estimation of the threshold value for KNN ceramics at the level of 300 nm. The other factor that influenced the value of dielectric permittivity is porosity—even a moderate porosity level can strongly depress the permittivity of the ceramic material. In the case of the discussed ceramic materials, the level of porosity does not change significantly, so it is difficult to clearly determine the degree of influence of pores concentration on dielectric properties.

The temperature dependencies of tan *δ* (Figure 6b) suggest that the prolongation from 25 h to 100 h of the mechanical activation time causes a significant reduction of the dielectric losses. Such a behaviour is very profitable from the application point of view. Recently, we observed a similar effect of the dielectric losses for mechanochemically activated BFN ceramics [35].

The second anomaly observed on the temperature dependence of dielectric permittivity is related to phase transition from the ferroelectric to the paraelectric phase. The anomalous behaviour is typical for ceramics obtained from powders after a long process of high energy milling. For classical ferroelectrics, permittivity follows the Curie–Weiss law above the Curie temperature:

(1) $$\varepsilon =\frac{C}{T-T_{0}},$$

where: *C* is the Curie–Weiss constant, *T*_0_ is the Curie–Weiss temperature.

**Figure 7:**
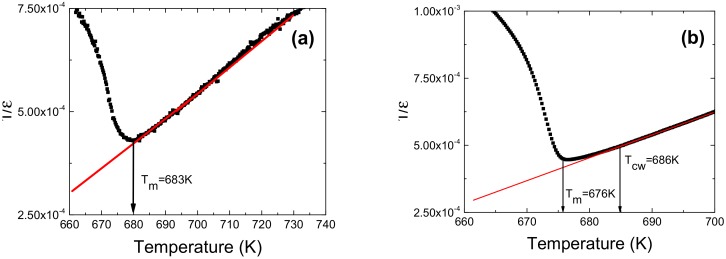
Inverse dielectric permittivity at 10 kHz as function of temperature for ceramics obtained from 25 h (**a**) and 100 h (**b**) milled powders (symbols: experimental data; solid line: fitting to Curie–Weiss law).

The reciprocal of dielectric permittivity as a function of temperature is shown in Figure 7 for ceramics obtained from the powders milled for 25 h and 100 h. The values of parameters *C* and *T*_0_ obtained as a result of the fitting procedure are summarized in Table 3.

The Curie-Weiss constant is related to the polar long range order. The higher value of *C* is correlated with a stronger order [5]. The comparison of *C* values of the investigated ceramics allows us to suggest that the stronger ferroelectricity occurs in ceramics obtained from 50 h milled powder.

In the case of the ceramics obtained from the powders milled for 75 h and 100 h, the deviations from the Curie–Weiss law are observed in a narrow range above *T_C_* and can be defined by Δ*T_D_* in the following way [36]:

(2) $$\Delta T_{D}=T_{CW}-T_{m},$$

*T_CW_* denotes the temperature from which the dielectric constant starts to follow the Curie–Weiss law and *T_m_* represents the temperature at which the dielectric permittivity reaches the maximum.

**Figure 8:**
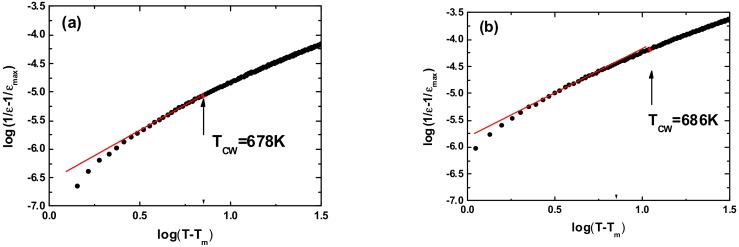
Log(1/*ε* – 1/*ε_max_*) as function of log(*T* − *T_m_*) at 10 kHz for KNN ceramics subjected to mechanosynthesis for a period of time equal to 75 h (**a**) and 100 h (**b**), symbols present experimental data; solid line—fitting to modified Curie–Weiss law.

For the ceramics activated by 75 h and 100 h, Δ*T_D_* is equal to 7 K and 10 K respectively. These values indicate that in both cases the phase transition has a diffuse character. The degree of diffuseness can be calculated from the modified Curie–Weiss law [37]:

(3) $$\frac{1}{\varepsilon }-\frac{1}{\varepsilon_{max}}=\frac{{\left(T-T_{max}\right)}^{\gamma }}{C_{1}},$$

where *ε_max_* is the maximum value of the dielectric permittivity at the transition temperature (*T_m_*), *C*_1_ is the constant, and *γ* is the degree of diffuseness. In the case when *γ* equals 1 the expression reduces to the Curie–Weiss law and is valid for a normal ferroelectric–paraelectric phase transition. Whereas, if *γ* equals 2 the quadratic dependence, valid for the ideal relaxor ferroelectric, is obtained. In order to obtain information about the diffuse phase transition behaviour, the plots of log(1/*ε* − 1/*ε_max_*) as a function of log(*T* − *T_m_*) at 10 kHz (Figure 8) were analysed for the KNN ceramics, obtained from the 75 h and 100 h activated powders.

**Figure 9:**
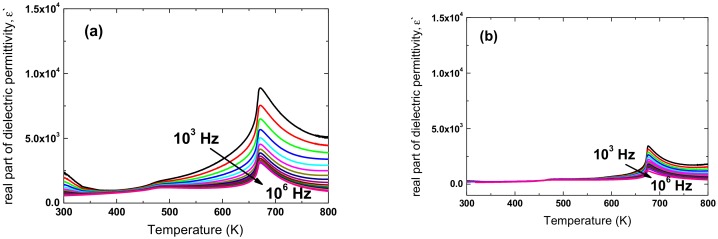
Dielectric permittivity as function of temperature measured at various frequencies of measuring field, for KNN ceramics obtained from 25 h (**a**) and 100 h (**b**) milled powders.

The linear relationship is observed in both ceramic samples. The slope of the fitting curves was used to determine the value of the diffuseness parameter. It was found that the *γ* value is equal to 1.65 and 1.69 for 75 h and 100 h activated ceramics, respectively. Such values of the *γ* parameter, which are connected with the wide character of the discussed maximum, could point to properties typical for ferroelectric relaxors. In order to verify this thesis, the measurements of the temperature dependencies of dielectric permittivity in the board range of frequency (500–10^6^ Hz) were performed. The results are presented in Figure 9, and do not confirm this assumption. Namely, in ferroelectric relaxors the characteristic dispersion is observed not only of the maximum value of dielectric permittivity, but also its temperature *T_m_*, which shifts with increasing frequency to a higher value. In the case of the investigated ceramics, the value of temperature *T_m_* remains unchanged.

## 4. Conclusions

Based on the presented investigation, the mechanochemical activation process has a significant impact on the final properties of the KNN ceramics. Moreover, the activation process lowers the reaction and sintering temperature. It can have a positive influence on the ceramics due to avoidance of grain growth. The milling duration affects the size of the grains. As a consequence, the dielectric properties are strongly dependent on the duration of the high energy milling. Also the temperature of the dielectric anomalies related to the phase transitions depends on the milling time. In comparison to conventionally prepared ceramics, the dielectric losses reach a lower level, whereas the dielectric permittivity of the ceramics obtained from the powders milled for 50 h and 75 h exhibit a higher value, up to 9200. Those materials are very promising candidates for further research in order to reduce pore concentration—it is commonly known that even a moderate porosity level can strongly depress the permittivity of the ceramic material [38].

## Figures and Tables

**Table 1:** Lattice parameters calculated for KNN ceramics obtained from t_M_ activated powders [17].

t_M_ (h)	Lattice Parameter			c_0_/a_0_	V (A^3^)
	a_0_ (A)	b_0_ (A)	c_0_ (A)		
25	3.965 (9)	5.635 (2)	5.654 (4)	1.425	126 (4)
50	3.963 (9)	5.626 (3)	5.667 (8)	1.429	126 (4)
75	3.962 (5)	5.619 (2)	5.672 (5)	1.432	126 (3)
100	3.967 (9)	5.627 (1)	5.676 (6)	1.431	126 (8)

**Table 2:** Value of dielectric permittivity in phase transitions and temperatures of phase transitions.

High-Energy Milling Time	Orthorhombic–Tetragonal Phase Transition		Ferroelectric–Paraelectric Phase Transition	
	T_OT_ (K)	ε_OTmax_	T_m_ (K)	ε_max_
25	480	553	679	2488
50	482	1340	676	9306
75	485	1358	673	4552
100	488	439	674	2225

**Table 3:** Curie–Weiss temperature *T*_0_, Curie–Weiss constant *C*, and diffuseness parameter *γ* for KNN ceramics at 10 kHz.

High-Energy Milling Time	*T*_0_ (K)	*C* × 10^5^ (K)
25	605	1.9
50	603	6.8
75	583	4.1
100	625	1.2
